# Deciphering the underlying mechanisms of Diesun Miaofang in traumatic injury from a systems pharmacology perspective

**DOI:** 10.3892/mmr.2015.3638

**Published:** 2015-04-16

**Authors:** CHUN-SONG ZHENG, CHANG-LONG FU, CAI-BIN PAN, HONG-JUAN BAO, XING-QIANG CHEN, HONG-ZHI YE, JIN-XIA YE, GUANG-WEN WU, XI-HAI LI, HUI-FENG XU, XIAO-JIE XU, XIAN-XIANG LIU

**Affiliations:** 1 Institute of Bone Disease, Academy of Integrative Medicine, Fujian University of Traditional Chinese Medicine, Fuzhou, Fujian 350122, P.R. China; 2 Department of Pharmacy, Xiamen Medical College, Xiamen, Fujian 361008, P.R. China; 3 Fujian Key Laboratory of Integrative Medicine on Geriatrics, Fujian University of Traditional Chinese Medicine, Fuzhou, Fujian 350122, P.R. China; 4 Beijing National Laboratory for Molecular Sciences, College of Chemistry and Molecular Engineering, Peking University, Beijing 100871, P.R. China

**Keywords:** traditional Chinese medicine, Diesun Miaofang, traumatic injury, polypharmacology, systems pharmacology

## Abstract

Diesun Miaofang (DSMF) is a traditional herbal formula, which has been reported to activate blood, remove stasis, promote qi circulation and relieve pain. DSMF holds a great promise for the treatment of traumatic injury in an integrative and holistic manner. However, its underlying mechanisms remain to be elucidated. In the present study, a systems pharmacology model, which integrated cluster ligands, human intestinal absorption and aqueous solution prediction, chemical space mapping, molecular docking and network pharmacology techniques were used. The compounds from DSMF were diverse in the clusters and chemical space. The majority of the compounds exhibited drug-like properties. A total of 59 compounds were identified to interact with 16 potential targets. In the herb-compound-target network, the majority of compounds acted on only one target; however, a small number of compounds acted on a large number of targets, up to a maximum of 12. The comparison of key topological properties in compound-target networks associated with the above efficacy intuitively demonstrated that potential active compounds possessed diverse functions. These results successfully explained the polypharmcological mechanism underlying the efficiency of DSMF for the treatment of traumatic injury as well as provided insight into potential novel therapeutic strategies for traumatic injury from herbal medicine.

## Introduction

Traumatic injury is one of the most common diseases in orthopedics. Its manifestations predominantly include localized pain, purplish swelling and joint dysfunction. Traumatic injury may also affect diet and sleep. The primary western drugs clinically available are analgesics and anti-inflammatory drugs, which predominantly alleviate the pain and swelling; however, these drugs have numerous side-effects (1). Recovery from traumatic injury is slow; therefore, it is essential to develop novel drugs suitable for prolonged use.

Diesun Miaofang (DSMF), a well-known herbal formula developed in the Ming Dynasty, has been widely used to treat traumatic injuries associated with parts of the body in China (2). The major herbs of this formula are *Angelica sinensis (AS)*, *Radix rehmanniae (RR)*, *Areca catechu (AC)* and *Radix paeoniae rubra (RPR)*; these herbs have been reported to work together to activate blood, remove stasis, promote qi circulation and relieve pain (3). However, the molecular features and active components of DSMF, as well as how these active components exert effects on targets remain to be elucidated. Several computer simulation methods have been successfully used to understand the theory of Traditional Chinese Medicine (TCM) and the relevant mechanisms of action from the molecular and systems level (4–8). In the present study, it was hypothesized that an integrated model combining cluster ligands, human intestinal absorption and aqueous solution prediction, chemical space mapping, molecular docking and network pharmacological techniques, may shed light on the herbal molecular features and pharmacological information of DSMF. These attempts offer key information for drug development from TCM and uncover holistic and synergic essence for TCM, which provided a guide for the modernization of TCM.

## Materials and methods

### Molecular database building and compound clustering

Chemical ingredients from *AS*, *RR, AC* and *RPR* were collected from the Chinese Herbal Drug Database (2002 version) and the Handbook of the Constituents in Chinese Herb Original Plants (9,10). Excluding duplicates, 158 compounds were determined and their structures were drawn using ISIS Draw 2.5 (MDL Information Systems, Inc., San Leandro, CA, USA), then further optimized using Discovery studio 2.0 (DS2.0; Accelrys, Inc., San Diego, CA, USA) with a Merck molecular force field (Merck Research Laboratories, Boston, MA,USA). The protocol of cluster ligands in DS2.0 were performed to group DSMF compounds under the standard default settings (11).

### Human intestinal absorption (HIA) and aqueous solubility prediction

HIA following oral administration and aqueous solubility were predicted using the ADMET module of DS2.0 (12). Compounds with an absorption level of 0, 1, 2 or 3 were considered to have good, moderate, poor or very poor absorption, respectively. Compounds with the solubility levels in the interval from 2 to 4 were considered to exhibit drug-like properties. Acceptable HIA means HIA level is 0 or 1, and acceptable solubility means solubility level values are 2, 3 or 4.

### Chemical space mapping and analysis

In order to investigate whether the four herbs had diverse chemical components, 150 physicochemical properties, including 1 dimensional (D)-, 2D- and 3D-molecular descriptors, were selected for analysis using the Quantitative Structure-Activity Relationship module of DS2.0 (6,13). Distribution of chemical ingredients in the chemical space was visualized via principal component analysis using the library analysis module of DS2.0. In addition, analysis of four important pharmacology-associated descriptors, including molecular weight (MW), number of HBond donors (nHDon), number of HBond acceptors (nHAcc) and octanol-water partition coefficients (AlogP), were performed to predict the drug-likeness of DSMF base on Sigmaplot version 10.0 (Systat Software, Inc., San Jose, CA, USA).

### Potential active compound prediction

Molecular docking was performed to determine whether DSMF interacted with the key proteins associated with blood circulation activation and anti-inflammation, using the LigandFit module of DS2.0. The crystal structures of protein-ligand complexes were downloaded from the Research Collaboratory for Structural Bioinformatics Protein Data bank (http://www.pdb.org/; Table I), with the exception of thromboxane A2 receptor (TP) (14–17). The homology model of TP was obtained from the Center for Experimental Therapeutics and Pharmacoinformatics (University of Houston, Houston, TX, USA)(18,19). For each crystal structure, crystallo graphic water molecules were removed and missing hydrogen atoms were added. The inhibitor from the crystal structure defined the active site. A total of 158 DSMF compounds were docked into the protein models and interactions between them were evaluated by DockScore which estimates the ligand position and orientation based on the most favorable energy production from the interactions between the ligand conformations and receptor proteins (20). Compounds with top 8 DockScores were selected as potential active compounds in DSMF (21).

### Network construction and analysis

To elucidate the functional mechanism underlying DSMF, the compound-target (C-T) network and herb-compound-target (H-C-T) network were constructed. The procedure for network construction was as follows: The C-T network was constructed by linking the potential active compounds and their corresponding targets; and the H-C-T network was constructed by connecting previously mentioned compounds to any associated herbs. All networks were produced and analyzed using Cytoscape 2.8.3 (University of California, San Diego, CA, USA) (22).

## Results

### Molecular features of DSMF

**Figure 1:**
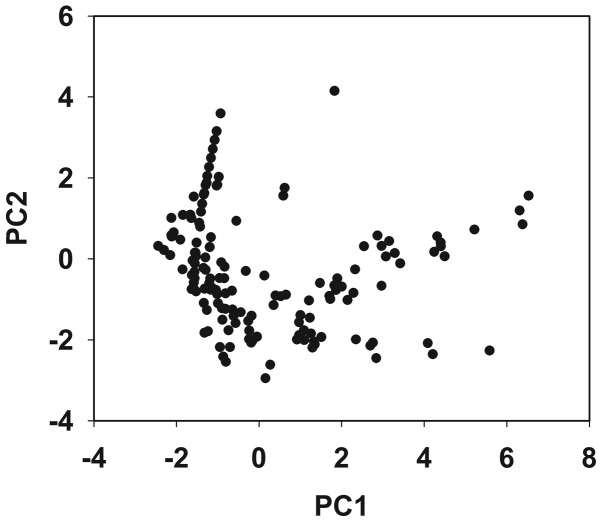
Chemical space distribution of compounds in Diesun Miaofang.

**Figure 2:**
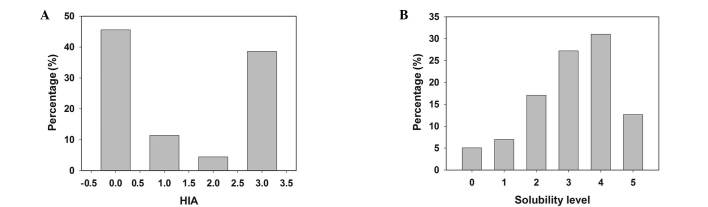
(A) Distributions of HIA and (B) aqueous solubility of compounds from Diesun Miaofang. HIA, human intestinal absorption.

**Figure 3:**
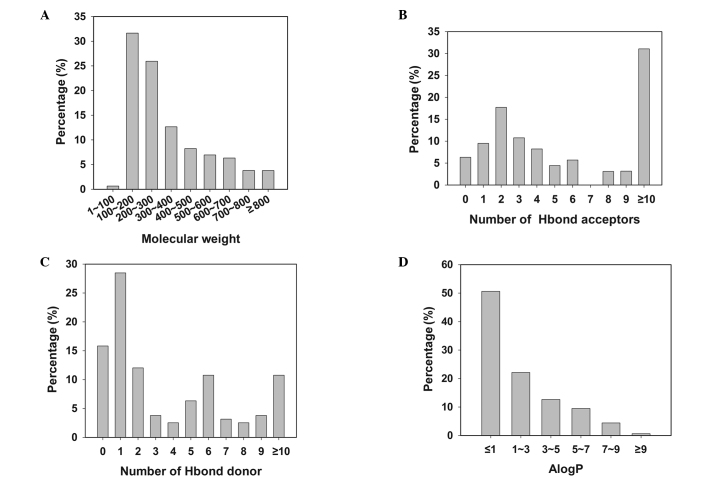
Profile distributions of four important molecular properties of compounds from Diesun Miaofang. The molecular properties consist of the molecular (A) weight, (B) number of HBond acceptors, (C) number of HBond donors and (D) AlogP. AlogP, octanol-water partition coefficients.

The compounds from DSMF were divided into 10 clusters (Table II). These compounds were widely distributed in the chemical space (Fig. 1; Table III). Additionally, >56% exhibited an acceptable HIA and aqueous solubility (Fig. 2). Four molecular descriptors, including MW, nHAcc, nHDon and AlogP, which reflect the basic characteristics of drug-likeness were analyzed and are shown in Fig. 3. The percentages of compounds with MW <500 Da, nHAcc <10, nHDon <5 and AlogP <5 were 79.12, 69.62, 62.66 and 85.44%, respectively. These results indicated that the compounds from DSMF were diverse and exhibited potent drug-like properties for oral administration, according to the ‘rule of five’ (23).

### Potential active compounds and diverse functions in DSMF

**Figure 4:**
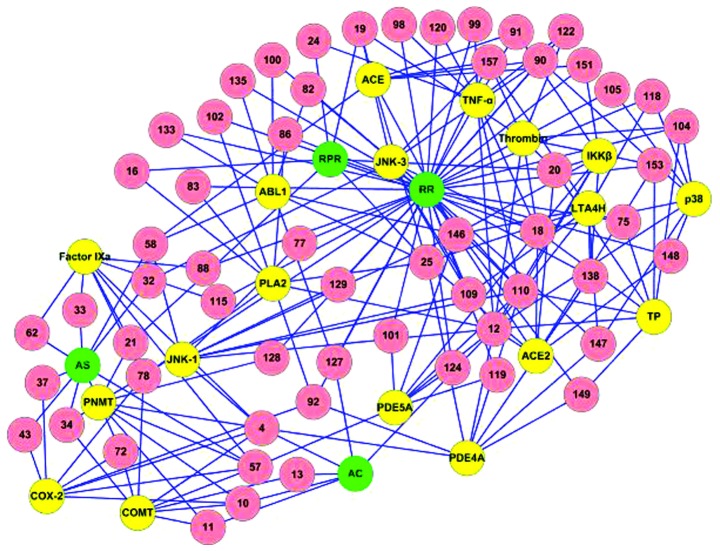
Herb-compound-target network. Green, pink and yellow circles represent the herbs, compounds and targets, respectively.

**Figure 5:**
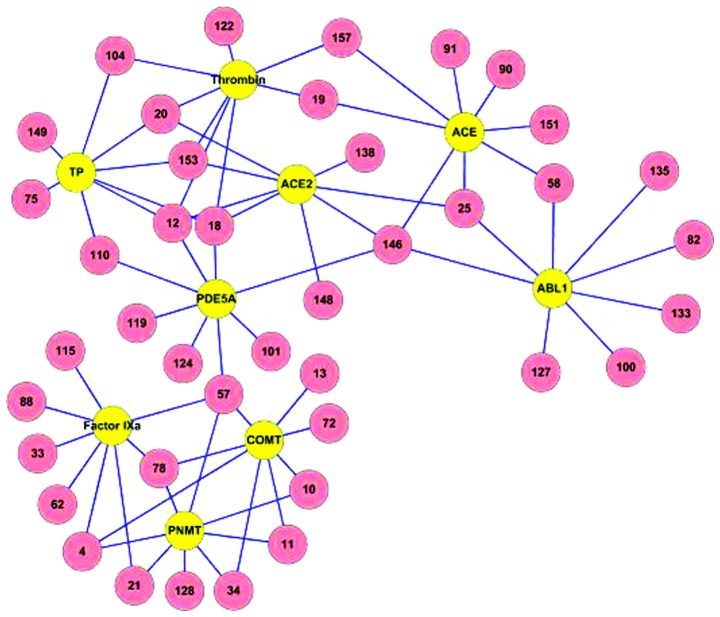
Compound-target network associated with activating blood and removing stasis. Pink and yellow circles represent compounds and targets, respectively.

**Figure 6:**
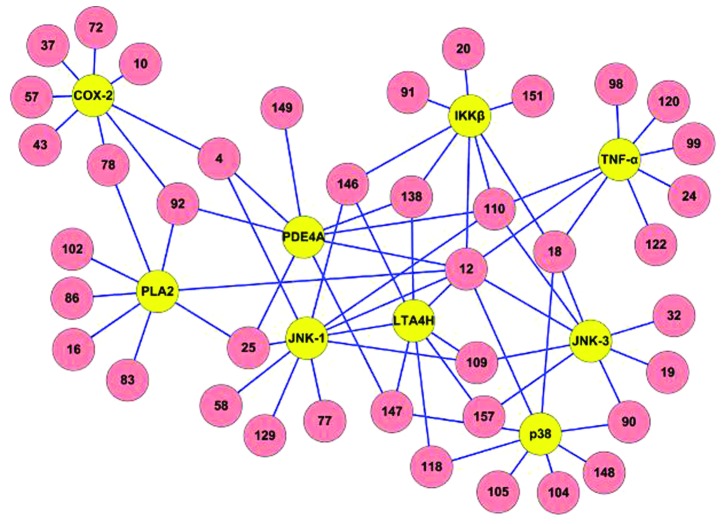
Compound-target network associated with promoting qi circulation and relieving pain. Pink and yellow circles represent compounds and targets, respectively.

**Figure 7:**
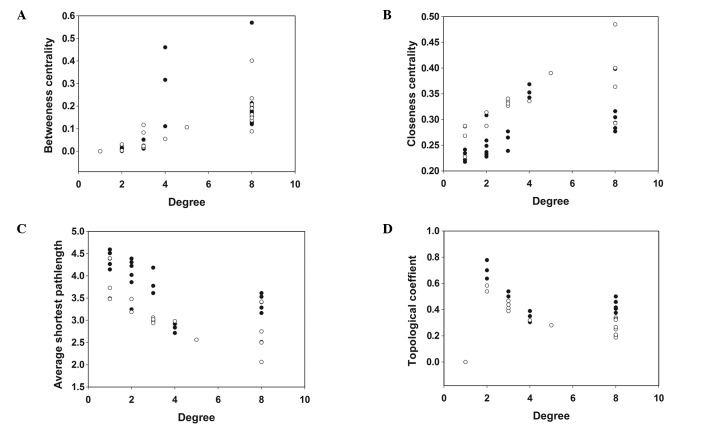
(A–D) Profile distributions of key topological properties for two compound-target networks. Black circles represent the topological properties of the compound-target network associated with activating blood and removing stasis and white circle represent those of the compound-target network associated with promoting qi circulation and relieving pain.

A total of 59 compounds were screened out by the docking method, which linked with compounds with 16 targets. To highlight the functions of DSMF, one H-C-T network and two C-T networks associated with activating blood, removing stasis, promoting qi circulation and relieving pain were constructed and are shown in Figs. 4–6. In Fig. 4, 29 compounds were associated with only one target, while 30 compounds of exhibited a relatively potent interaction with ≥2 targets. Notably, six compounds were associated with >5 targets. The key network parameters of the above networks and compounds in the H-C-T network were listed in Tables IV and V, respectively. The topological properties of two C-T networks were analyzed in Fig. 7. The two C-T networks with different functions had a small number of overlaps in the distributions of betweenness centrality, closeness centrality, average shortest path length and topological coefficients, which indicated that certain compounds in the two networks may have synergy, and more compounds may possess diverse functions.

## Discussion

TCM, as an important part of complementary and alternative medicine, demonstrated an improved effect on osteopathia (24). However, the active substances involved in the herbal formulae and the mechanisms of action associated with the therapeutic effectiveness remain to be fully elucidated. Additionally, conventional methods of herbal investigation generally focus on extraction, isolation, purification, structure identification and activity assays. These often lead to a failure to screen out certain active ingredients from herbs and may not reflect the integrality of TCM (25). Therefore, determining reasonable approaches to solve the above shortcomings has become a focus of research (26,27).

There has been an increasing focus on identifying novel drugs from TCM and elucidating the underlying pharmacological mechanisms of TCMs via different modern computational pharmacological methods (28). These are important for improving current understanding of the nature of TCM. In the present study, Table II demonstrated that the compounds from DSMF may be separated into 10 independent clusters. The compounds from clusters 1, 2, 3, 4, 8, 9 and 10, were observed in no less than two herbs, suggesting that different herbs may have similar structural features. The clusters 5, 6 and 7 also suggested that different herbs may have their own structural features. In order to gain more knowledge regarding the physicochemical features of compounds from DSMF, the chemical space of DSMF was mapped in Fig. 1. According to the theory of chemical space (13), the superposed and close dots indicated that these compounds may have similar mechanisms of action and may act on identical targets. Conversely, dispersive dots indicated that these compounds may have different pharmacological activities. These results may explain why DSMF has diverse efficacies from TCM theory. In addition, Figs. 2 and 3 demonstrated that the compounds from DSMF have favorable drug-like properties and are suitable for screening lead compounds.

Docking results revealed that 59 potential compounds exhibited desired interactions with the targets. The global understanding of the association among the herbs, compounds and targets was shown in Fig. 2. Out of the 59 potential compounds, 30 had at least two links with other targets, indicating that the majority of targets share common compounds with other targets. The average number of potential targets per compound was 3.28. In addition, the four herbs from DSMF were found to work together on 9 common targets, suggesting that DSMF was a broad-spectrum formula acting on multiple targets. Since the synergistic multitarget effects mean that natural products affect not only one target, but several targets (5), DSMF has potential synergistic interactions.

To further elucidate the multicompound therapeutic mechanism of DSMF in the present study, the key topological properties of two compound-target networks associated with activating blood, removing stasis, promoting qi circulation and relieving pain, including betweenness centrality, closeness centrality, average shortest path length and topological coefficient, were selected to perform the analysis (Figs. 5, 6 and 7). These results may provide important information with regards to the properties of compounds and proteins involved in the network. As shown in Fig. 5, the efficacy of activating blood and removing stasis may be accomplished by interacting with several targets associated with hemostasis and blood circulation. Fig. 6 also demonstrated that DSMF may promote qi circulation and relieve pain by influencing several targets associated with anti-inflammation. For example, ferulic acid in Table V presents a relatively strong interaction with these targets associated with hemostasis and blood circulation (catechol-O-methyl transferase, phenylethanolamine N-methyltransferase, phenyl-ethanolamine, phosphodiesterase 5A and Factor-IXa) and anti-inflammation (cyclooxygenase-2). Notably, it has been reported that ferulic acid had effects on anti-platelet aggregation and alleviating pain (29,30). In addition, in the present study, Fig. 7 demonstrated that certain nodes with an identical degree may have different effects on the above topological properties, which suggested that there are different modes of action between compounds and targets during the treatment of DSMF. Taken together, these results consistently indicated that DSMF exists as multicompounds and combination therapies to act on several targets, finally exerting diverse pharmacological effects on traumatic injury.

In conclusion, the present study provided a systems pharmacology model to dissect the molecular features and modes of action of DSMF. The key findings demonstrated that the compounds from DSMF were diverse in chemical structure and widely distributed in chemical space, the majority of the compounds from DSMF had potent drug-like properties for oral administration and DSMF possessed multicompound synergy associated with the interacting targets and exerted different pharmacological effects. The present study provided a systematic view of the association between components in herbal formula, the efficacy of herbal formula and targets, which contributed to an improved understanding of the chemical and pharmacological basis of TCM, which may enhance the process of drug discovery from TCM.

## Figures and Tables

**Table I:** Key target proteins associated with activating blood, removing stasis, promoting qi circulation and relieving pain.

A, Targets associated with activating blood and removing stasis	
Protein	PDB code
Thrombin	1AWH
Factor-IXa	1RFN
PDE5A	4G2W
ACE	1O86
ACE2	1R42
Catechol-O-methyl transferase	3BWM
Phenylethanolamine N-methyltransferase	2AN4
Abelson tyrosine-protein kinase1	3QRI

B, Targets associated with promoting qi circulation and relieving pain	
Protein	PDB code
Inhibitor of nuclear factor κ-B kinase-β	3RZF
JNK-1	2GMX
JNK-3	1PMN
P38	1A9U
Cyclooxygenase-2	6COX
Phospholipase 2	1DB4
Leukotriene A4 hydrolase	3B7R
Tumor necrosis factor-α	2AZ5
PDE4A	3TVX

PDB, protein data bank; PDE, phosphodiesterase; ACE, angiotensin-converting enzyme; JNK, c-Jun N-terminal kinases; TNF, tumor necrosis factor.

**Table II:** Cluster result of compounds from Diesun Miaofang.

Cluster	Number of compounds	Source
1	7	*AS, RR, AC*
2	13	*AS, AC*
3	34	*AS, RR, AC*
4	28	*RR, AC, RPR*
5	2	*RR*
6	2	*AS*
7	2	*AC*
8	14	*AS, RR, AC, RPR*
9	13	*AS, RR, AC*
10	43	*RR, RPR*

AS, Angelica sinensis; RR, Radix rehmanniae; AC, Areca catechu; RPR, Radix Paeoniae Rubra.

**Table III:** Key molecular properties of compounds in Diesun Miaofang.

Property	Mean	Standard deviation	Minimum	Maximum
Number of carbon atoms	16.67	8.97	4	41
Number of nitrogen atoms	0.16	0.79	0	8
Number of oxygen atoms	6.66	6.33	0	26
Molecular weight	334.78	205.36	94.11	940.68
Number of hydrogen acceptors	6.78	6.31	0	26
Number of hydrogen donors	3.78	3.91	0	17
Octanol-water partition coefficients	1.07	3.45	−9.55	9.13

**Table IV:** Simple parameters of H-C-T and C-T networks.

Parameter	H-C-T network	C-T network function	
		Activating blood and removing stasis	Promoting qi circulation and relieving pain
Network centralization	0.423	0.109	0.110
Network heterogeneity	0.911	0.891	0.926
Characteristic path length	2.821	3.924	3.372
Average number of neighbors	5.012	2.880	2.939

H-C-T, herb-compound-target; C-T, compound-target.

**Table V:** Key compounds in the herb-compound-target network.

Degree	Index	Chemical name
12	12	Proanthocyanidin B2
8	18	Eugeniin
7	146	Rehmannioside A
7	110	Isoacteoside
6	25	(Z)-(1S,5R)-β-Pinen-10-yl-β-vicianoside
6	4	Catechin
5	78	Adenosine
5	57	Ferulic acid
4	157	6-O-Vanilloylajugol
4	20	Oxypaeoniflorin

## Abbreviations

DSMF: Diesun Miaofang
*AS*: *Angelica sinensis*
*RR*: *Radix rehmanniae*
*AC*: *Areca catechu*
*RPR*: *Radix paeoniae rubra*
TCM: Traditional Chinese Medicine
HIA: human intestinal absorption
PDE5A: phosphodiesterase 5A
PDE4A: phosphodiesterase 4A
ACE: angiotensin-converting enzyme
COMT: catechol O-methyltransferase
PNMT: phenylethanolamine N-methyltransferase
IKK: inhibitor of κB kinase
JNK: c-Jun terminal kinase
COX-2: cyclooxygenase-2
PLA2: phospholipase A2
LTA4H: leukotriene A-4 hydrolase
TNF-α: tumor necrosis factor-α
TP: thromboxane A2 receptor
MW: molecular weight
nHDon: number of HBond donors
nHAcc: number of HBond acceptors
AlogP: octanol-water partition coefficients
Std Dev: standard deviation
